# The impact of torasemide on haemodynamic and neurohormonal stress, and cardiac remodelling in heart failure – TORNADO: a study protocol for a randomized controlled trial

**DOI:** 10.1186/s13063-016-1760-z

**Published:** 2017-01-23

**Authors:** Paweł Balsam, Krzysztof Ozierański, Agata Tymińska, Renata Główczyńska, Michał Peller, Anna Fojt, Andrzej Cacko, Bartosz Sieradzki, Elwira Bakuła, Maciej Markulis, Robert Kowalik, Zenon Huczek, Krzysztof J. Filipiak, Grzegorz Opolski, Marcin Grabowski

**Affiliations:** 1st Chair and Department of Cardiology, Medical University of Warsaw, Public Central Teaching Hospital in Warsaw, 1a Banacha St., Warsaw, 02-097 Poland

**Keywords:** Dose, Furosemide, Heart failure, Hospitalization, Loop diuretic, Prognosis, Quality of life, Randomized controlled trial, Torasemide

## Abstract

**Background:**

Approximately 50% of heart failure patients are readmitted to hospital within 6 months, owing to deterioration of their condition. Thus, symptomatic treatment of heart failure requires significant improvement. The aim of this study is to compare the effects of torasemide and furosemide on biochemical parameters of haemodynamic and neurohormonal compensation, myocardial remodelling, clinical outcomes and quality of life in patients with chronic heart failure.

**Methods:**

This is a multicentre, randomized, open, blinded endpoint phase-IV trial. The study includes 120 heart failure patients in NYHA (New York Heart Association) functional class II–IV, treated with optimal heart failure therapy, with indications for use of loop diuretics. At enrolment, patients are stable, with a fixed dose of loop diuretics. Patients are randomized to treatment with furosemide or torasemide (randomization 1:1). After randomization, the current fixed dose of furosemide is continued or is replaced by an equipotential dose of torasemide (4:1). The study consists of two control visits (3 and 6 months after enrolment) with minimal follow-up of 6 months. Assessment involves clinical examination, Quality of Life Questionnaire, laboratory tests, echocardiography, electrocardiography, 24 h Holter-electrocardiography monitoring, 6 -min walk test and assessment of fluid retention. Any need for dose adjustment is assessed during the observation. The primary objective is to compare the effects of torasemide and furosemide on clinical and biochemical parameters of haemodynamic and neurohormonal compensation and myocardial remodelling. Secondary objectives include monitoring of: changes in signs and symptoms of heart failure, NYHA functional class, quality of life, dosage changes, rate of readmissions and mortality.

**Discussion:**

Despite decades of the diuretic’s history, knowledge about diuretic therapy is still unsatisfactory. The most widely used diuretic, furosemide, has a stormy pharmacokinetics and pharmacodynamics, and is associated with a high risk of mortality and hospitalization for worsening heart failure. Reports are very encouraging and suggest beneficial effects of torasemide. Hence, there is a need for further studies of the overall effect of torasemide, compared with furosemide. This can translate into improved quality of life and better prognosis of patients with heart failure.

**Trial registration:**

ClinicalTrials.gov, NCT01942109. Registered on 24 August 2013.

**Electronic supplementary material:**

The online version of this article (doi:10.1186/s13063-016-1760-z) contains supplementary material, which is available to authorized users.

## Background

In developed countries, about 2% of an adult population have heart failure, with the incidence increasing to ≥10% among persons aged ≥70 years [1]. Despite continuous improvement in therapy, heart failure remains a condition with a poor prognosis. Mortality in heart failure reached 50% in a 5 year observation [1]. Decompensated heart failure is also the most common cause of hospitalization of adults [1]. Approximately 50% of patients with heart failure are readmitted to hospital within 6 months of discharge, owing to deterioration of their condition [1].

Angiotensin-converting enzyme inhibitors and β-blockers improve long-term outcomes in patients with heart failure; according to current guidelines, these drugs are the first-line treatment in heart failure patients [1]. The main manifestations of heart failure worsening are dyspnoea, reduced exercise tolerance, peripheral oedema, orthopnoea, jugular venous distension and increased nocturnal diuresis [1]. To improve symptoms and exercise capacity in patients with heart failure, diuretics are recommended [1]. The number of readmissions related to heart failure can be reduced by proper management of fluid retention [2]. Meta-analysis revealed that diuretic-based treatment, compared with placebo, could decrease hospitalizations for worsening of heart failure, as part of symptomatic treatment [3, 4]. Current guidelines do not emphasize superiority of any of the available diuretics [1]. In practice, the most commonly used diuretic in patients with heart failure is a loop diuretic – furosemide. Importantly, furosemide has no positive effect on patients’ outcomes and may even be associated with increased risk for hospitalization and all-cause and cardiovascular mortality [5]. Therefore, there is a need to seek a safer and more effective drug than furosemide.

A drug with greater potential seems to be torasemide, but so far, there is a very poor record of research into its participation. It is postulated that, compared with furosemide, torasemide reduces readmissions due to heart failure [6]; moreover, there is a discussion of its possible beneficial effect on mortality [7]. Yet torasemide has been shown to have less inter- and intra-individual variation in bioavailability and longer action than furosemide [8]. Vargo *et al.* reported that heart failure does not affect the rate of absorption of orally administered torasemide, in contrast with furosemide, for which absorption was delayed [9]. Thus, torasemide has more predictable pharmacokinetics and pharmacodynamics and lower influence on electrolyte disorders and therefore has improved tolerability compared with furosemide [10]. Torasemide also induces greater improvements in functional and social limitations. Some clinical studies showed improvement in New York Heart Association (NYHA) functional class and pulmonary haemodynamics, as well as reduced body weight, in patients who received torasemide [8, 11, 12]. In a study by Müller *et al.*, torasemide decreased the number of mictions at 3, 6 and 12 h after diuretic intake, as well as the urgency to urinate [11].

Diminished cardiac output, seen in heart failure, provokes the release of vasopressin and activates a renin-angiotensin-aldosterone system [13]. Consequently, oedemas are produced through increased sodium and water retention [13]. Some studies showed unfavourable influence of furosemide on the renin-angiotensin-aldosterone system [14, 15]. In contrast, torasemide combines the effects of both furosemide and spironolactone [16]. Therefore, torasemide may also have an anti-androgenic effect; however, it is not fully understood whether torasemide acts through a mineralocorticoid receptor or not [16]. It has been reported that torasemide may block the renin-angiotensin-aldosterone system [17] and might therefore attenuate myocardial remodelling accompanied by left ventricular dysfunction [18]. Simultaneously, Lopez *et al.* related the use of torasemide with decrease in cardiac fibrosis in biopsy specimens from hypertensive patients with chronic heart failure [19]. Those additional pleiotropic effects could make torasemide more beneficial than furosemide.

Additionally, torasemide could be a cost-saving option compared with furosemide. Pharmacoeconomic analysis shows that it reduces the overall cost of in-hospital [20] and long-term treatment of chronic heart failure through the reduction of hospital admissions [21]. Treatment with torasemide showed 80% reduction of in-hospital days and 30% decrease in lost working days, compared with furosemide [22].

Only direct comparison of furosemide and torasemide could present the similarities and differences of these two agents. Available reports presented clinical and economic advantages of torasemide, and at least two expert groups recommended considering torasemide use first over furosemide in heart failure patients [23, 24]. However, more long-term data are needed to confirm these results and to investigate the effect of torasemide on quality of life. This report aims to describe a randomized clinical trial protocol designed to compare the effectiveness of torasemide versus furosemide in improving cardiac remodelling, haemodynamic stress and neurohormonal stress. Additionally, the trial aims to assess the clinical stability, one-year readmission rate and mortality in patients with heart failure treated with torasemide compared with furosemide. The hypothesis of this study is that torasemide may present more favourable effects than furosemide on biochemical and clinical parameters (e.g. biochemical biomarkers, clinical symptoms, quality of life, long-term outcomes) in patients with heart failure.

## Methods/design

### Study objectives

The primary objective of the study is to compare the effects of torasemide and furosemide on clinical and biochemical parameters of haemodynamic and neurohormonal compensation, as well as myocardial remodelling in patients with chronic heart failure with indications for use of loop diuretics.

Secondary objectives include the comparison of torasemide and furosemide in relation to changes in signs and symptoms of chronic heart failure, an improvement in NYHA functional class and quality of life, a need for dosage changes, as well as rate of readmissions and mortality in long-term observation.

### Study design

**Fig. 1:**
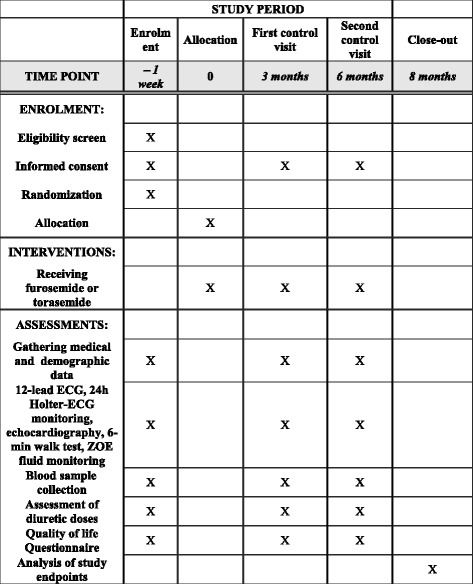
Standard Protocol Items: Recommendations for Interventional Trials (SPIRIT)

The study is an investigator-initiated, multicentre, randomized, open, blinded endpoint phase-IV trial with parallel groups. The study is being carried out in two tertiary university hospitals on cardiology wards. The study will include 120 hospitalized patients with heart failure. Approval was obtained from institutional review boards. All participants must sign an informed consent form. To reach the adequate target sample size, a list of new admissions of patients with heart failure is checked every day. The study protocol conforms to the Standard Protocol Items: Recommendations for Interventional Trials (SPIRIT) 2013 statement (Fig. 1) [25]. See Additional file 1 containing the SPIRIT Checklist.

### Study population

Patients included in the study are hospitalized and have chronic heart failure in NYHA functional class II–IV. The diagnosis of heart failure, according to current guidelines [1], is based on clinical (typical heart failure signs and symptoms), echocardiographic and biochemical (increased concentrations of N-terminal pro-B-type natriuretic peptide or B-type natriuretic peptide) parameters. Patients are treated with optimal heart failure therapy and have indications for use of loop diuretics to maintain euvolaemia. At the time of enrolment patients are in stable condition or with exacerbation-aligned cardiopulmonary symptoms, with a fixed dose of loop diuretics.

### Inclusion and exclusion criteria

Patients meeting all inclusion criteria and no exclusion criteria, as shown in Table 1, are enrolled in the study.

**Table 1:** Inclusion and exclusion criteria

Inclusion criteria:
Signed informed consent form
Age ≥18 years
Patients with chronic heart failure, as defined by the European Society of Cardiology (update 2016)
New York Heart Association (NYHA) functional class II–IV
Patients who require diuretic therapy for maintain euvolaemia
Stable clinical condition during index hospitalization with established proper diuretic dosage
Exclusion criteria:
Acute coronary syndrome
Hypertrophic cardiomyopathy
Uncontrolled hypertension
Uncontrolled diabetes
Serum potassium >6.0 mmol/l
Serum creatinine >2.5 mg/dl
Malignancy
Life expectancy <1 year
Planned valve surgery or heart transplantation

Reasons for discontinuing or modifying allocated diuretics are noted (i.e., drug dose change in response to harms, participant request, or improving or worsening disease).

### Overall study description

**Fig. 2:**
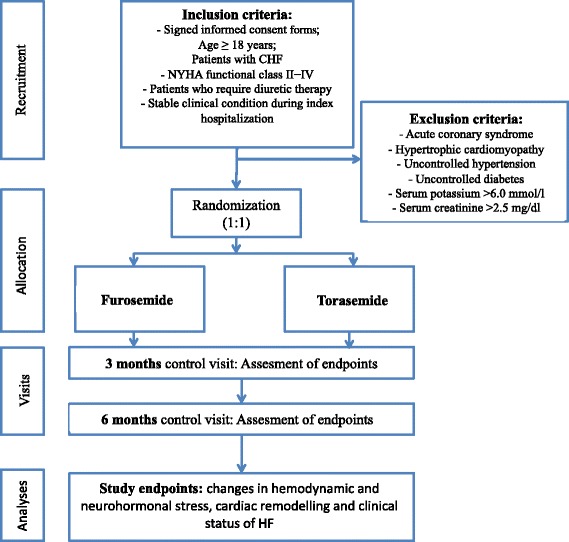
Study design as flow diagram: recruitment, randomization, intervention, follow-up, assessment and outcomes. CHF, chronic heart failure; NYHA, New York Heart Association

During hospitalization, patients with chronic heart failure who meet the inclusion criteria are asked to participate in the study. A summary of the study course is presented in Fig. 2. Assumptions of the study are explained and informed written consent is obtained. A case report form is created for each patient. Researchers gather data regarding medical history, demographics, clinical presentation, previous and current treatment, diagnostic test results and the clinical course of index hospitalization. During hospitalization, 12-lead electrocardiography, 24 h Holter-electrocardiography monitoring, echocardiography, a 6 min walk test, and assessment of fluid retention using a ZOE fluid status monitor are performed, and blood sample is obtained for further measurements of biomarkers. Patients are also asked to complete the Quality of Life Questionnaire (SF-36v2).

Patients are randomized by investigators to treatment with furosemide or torasemide (randomization 1:1). Patients receive a number from 001 to 120. After randomization, the current fixed dose of furosemide is continued or is replaced by an equipotential dose of torasemide (4:1, according to the producer’s recommendations). Patients receive the proper diuretic (furosemide or torasemide) in suitable doses at a baseline hospitalization and then at control visits after 3 and 6 months. The short duration of treatment, frequent control visits and ease of study drug administration facilitate adherence. To maximize compliance, retention in the study and adherence to treatment, patients are monitored prospectively and routinely throughout the trial with frequent telephone contact.

The study consists of two control visits (3 and 6 months after enrolment) with a minimal follow-up period of 6-months. During control visits, changes in clinical and biochemical status compared with the baseline are monitored. Clinical examination is performed for signs and symptoms of heart failure (dyspnoea, orthopnoea, urinary frequency, peripheral oedema, S3 heart tone, jugular venous distension, pulmonary rales, NYHA functional class), assessment of exercise tolerance (6 min walk test) and fluid retention (by measurement of a body weight and body impedance monitor; the ZOE fluid status monitor). Based on these tests, investigators may decide to continue the previously established drug dose or change the dosage. Additionally, the Quality of Life Questionnaire (SF-36v2) will be completed, blood samples will be obtained, and echocardiography, 12-lead electrocardiography and 24 h Holter-electrocardiography monitoring will be performed. Blood samples collected during baseline hospitalization and control visits are stored in a laboratory of the university. All blood samples will be analyzed after the end of enrolment.

### Study endpoints

Adverse events appearing after signing the informed consent are documented. The study endpoints consist of comparison of torasemide versus furosemide in relation to changes in haemodynamic and neurohormonal stress, cardiac remodelling and clinical status of heart failure from baseline through the follow-up.

Interactions of loop diuretics with specific subgroups will be assessed. In particular, the investigators will provide an additional analysis of influence of diuretics on outcomes in clinically relevant subgroups, i.e. patients with implantable devices, elderly and dilated cardiomyopathy.

### Assessment of outcomes

#### Haemodynamic and neurohormonal stress

Biochemical (N-terminal pro-B-type natriuretic peptide, suppression of tumorigenicity 2 (ST2)), clinical (heart rate, arterial blood pressure) and echocardiographic (left ventricular ejection fraction, pressures in cardiac chambers) parameters, considered to be associated with increased haemodynamic and neurohormonal stress [26, 27] are measured. Blood samples are collected and echocardiographic studies are performed at the baseline and control visits.

#### Cardiac remodelling

Cardiac remodelling is evaluated in terms of changes in concentrations of biomarkers and echocardiographic parameters. Biomarkers responsible for myocardial fibrosis, necrosis and inflammation (Galectin-3, ST2, high-sensitive troponin T, collagen peptides, metalloproteinases) are considered [28–30]. During echocardiographic study, the left ventricular end-diastolic dimension, left ventricular end-systolic dimension, intraventricular septum and posterior wall thickness, left ventricular mass index, left ventricular ejection fraction, valve flows, and right ventricular and atrial dimensions will be measured). Blood samples are collected and echocardiography is performed at the baseline and control visits.

#### Clinical status of heart failure

An important element of the study is to evaluate changes in clinical status of heart failure and outcomes in long-term observation, in both groups – furosemide and torasemide. The composite endpoint measures include changes in signs and symptoms of chronic heart failure (heart rate, arterial blood pressure, NYHA functional class change, distance covered in 6 min walk test, electrocardiography recordings). Additional endpoint measures are need for intensification of diuretics dosage, changes in laboratory tests (ionogram, renal function, biochemical profile), rate of readmissions or death owing to heart failure worsening, presence of other adverse events (i.e. myocardial infarction, thromboembolic events), changes in quality of life (based on the Quality of Life Questionnaire (SF-36v2) [31]), the presence or exacerbation of atrial or ventricular arrhythmias (12-lead electrocardiography, 24 h Holter monitoring) and assessment of fluid retention (presence of peripheral oedema, measurement of body weight and body impedance). Body impedance is measured using a ZOE fluid status monitor, which is a device determining the level of fluid retention on the basis of the resistance (impedance in ohms) of the human body. The less the impedance, the more hydrated is the body. The same model of case report form is used at baseline and follow-up visits.

### Data management and archiving

All data are coded and stored on university password-protected computers. Any physical copies of the case report forms and all study-related documents are archived at the university. All data are checked monthly by investigators (MG, KJF, GO) to ensure that all protocols and ethical guidelines for data collection and analysis are followed.

### Power analysis

The study is designed to detect a 50% reduction of primary endpoint prevalence between the groups. Under the assumption that death and rehospitalization are relatively common in patients with heart failure, we estimate the frequency of primary endpoint in the furosemide group as 60% [32]. Sample size calculation was performed for 5% type I error rate with 70% power.

The total number of events needed in study group to attain 70% power with an alpha of 0.05, is 54. To achieve this number of events, we plan to recruit 120 patients.

### Statistical analysis

Fisher’s exact test and the Mann–Whitney *U* test will be performed for comparison of both groups, for categorical variables and continuous variables, respectively. To determine the risk factors of primary and secondary endpoints, logistic regression analysis or the Cox proportional hazards regression model will be performed. To assess the independent influence of study drug on the outcome, multivariate logistic regression analysis will be performed. Statistical significance will be considered for *P* < 0.05 for all tests. Statistical analysis will be performed using SAS software, version 9.4.

### Limitations of the study

The study is not a double-blind trial; this might have some impact on the results. Moreover, the small number of participants will probably not enable assessment of the impact of torasemide and furosemide in different clinically relevant subgroups i.e. elderly, patients with chronic kidney disease, dilated cardiomyopathy. The relatively small sample size might not show the clinical benefits associated with torasemide for hard cardiovascular events. However, if an improvement in clinical condition and fluid retention is demonstrated, we feel that this might have a positive effect in the treatment of heart failure patients.

### Data monitoring and auditing

Study participants are closely monitored by the investigators. Case report forms are used to record any suspected harms of the study treatment. Study participants may be withdrawn from the study if there is suspicion of treatment harm (e.g. worsening renal function, allergic reaction). Monitoring of the data and quality of the study is performed by the senior-investigators (GO and KJF) who compose a data monitoring committee.

## Discussion

The currently available data suggest that torasemide may be a better alternative for treatment of congestion in chronic heart failure than furosemide.

However, answers to many questions about the superiority and usefulness of torasemide are still missing. The hypothesis of this study assumes that torasemide may present more favourable effects than furosemide on biochemical and clinical parameters of haemodynamic stress, cardiac remodelling, as well as clinical symptoms, quality of life and long-term outcomes in patients with heart failure.

If a reduction in haemodynamic and neurohormonal stress, as well as cardiac remodelling is demonstrated, this may prove additional advantages of torasemide compared with furosemide. This study might add valuable information about torasemide, supporting its more frequent use.

## Trial status

At the time of the manuscript submission, 50 patients were enrolled in the study and 16 of them had finished the follow-up. We expect to finish the study in 2017.

## Additional file

Additional file 1: SPIRIT 2013 checklist. (DOC 123 kb)

## Abbreviations

NYHA: New York Heart Association
SPIRIT: Standard Protocol Items: Recommendations for Interventional Trials
ST2: suppression of tumorigenicity 2
